# Implant removal: benefits and drawbacks - Results of a survey with five hundred participants from the Italian Society of Orthopedic Surgery and Traumatology (SIOT) and comparison with other international trends

**DOI:** 10.1007/s00264-025-06564-7

**Published:** 2025-05-26

**Authors:** Virginia Masoni, Corrado Ciatti, Luca Andriollo, Giovanni Vicenti, Fabrizio Rivera

**Affiliations:** 1 University of Turin, Turin, Italy; 2 Guglielmo da Saliceto Hospital, Piacenza, Italy; 3 University of Parma, Parma, Italy; 4 Fondazione Poliambulanza Istituto Ospedaliero, Brescia, Italy; 5 Catholic University of the Sacred Heart, Milan, Italy; 6 University of Bari Aldo Moro, Bari, Italy; 7 Ospedale SS Annunziata, ASL CN1, Savigliano, Italy

**Keywords:** Implant removal, Survey, Hardware, Asymptomatic, Stripping screw, Complications, Titanium

## Abstract

**Introduction:**

Implant removal in orthopedics and traumatology is still a controversial topic. Benefits and drawbacks lead to relative indications, mainly depending on patients’ demands and surgeons’ perspectives. This study aims to report the current attitudes and practices of Italian surgeons who participated in a survey.

**Materials and Methods:**

An online-based questionnaire of 25 items was distributed to all the members of the Italian Society of Orthopedic Surgery and Traumatology (SIOT) regarding their indications, usual practices, and complications encountered with hardware removal in upper and lower limbs. The survey was open from July 2024 to October 2024. Exclusion and inclusion criteria were applied.

**Results:**

Five hundred answers were received. While implant removal is primarily achieved in symptomatic patients, in the case of asymptomatic patients, it is not routinely performed, with a slightly higher tendency of removal in those aged 16–40 years old. These tendencies were registered both for the upper and lower limbs, with more reticence in hardware removal in the upper limbs. 96% of respondents declared the lack of hospital guidelines regarding this kind of surgery. The most feared intraoperative complications during the removal concerned screw stripping and implant breakage, with only 0.6% of respondents reporting no intraoperative difficulties. While patient discomfort and avoidance of future complications were the main indications for removal, postoperative complications occurred as wound scarring concerns, persistence of symptoms and bleeding. Despite not being considered a “procedure for the resident”, when residents were specifically questioned, in 76% of cases they felt self-confident ≥ 7 on a scale from 1 to 10. Lastly, according to 62% of the respondents, titanium implants are more difficult to remove than stainless steel ones.

**Conclusion:**

This survey describes a general tendency to not routinely remove implants, even in younger patients in the lower and especially upper limbs, unless in case of symptoms. Hardware removal could evolve from a simple procedure into a more complex surgery due to intraoperative technical difficulties. A lack of universal policy and guidelines exists throughout the Italian territory.

**Supplementary Information:**

The online version contains supplementary material available at 10.1007/s00264-025-06564-7.

## Highlights

- A general tendency of not routinely removing the implants in asymptomatic patients emerged, even in younger patients aged 16-40 years old.
- In the case of symptomatic patients, implant removal is considered a therapeutic possibility.
- Implant removal could evolve from a simple to a complex procedure since implants differ in materials, types, and positions.
- A lack of evidence-based guidelines exists throughout the Italian scenario.

**Supplementary Information:**

The online version contains supplementary material available at 10.1007/s00264-025-06564-7.

## Introduction

The number of surgeries for implant removal varies from country to country, with the percentages ranging from approximately 5–30% of all elective orthopaedic procedures [1–4]. This heterogeneity appears dictated by the lack of evidence-based guidelines [2–4]. Indeed, while there are circumstances such as in case of symptoms, functional deficit, infection, implant failure/migration, nonunion and malunion where implants are routinely removed [2, 5, 6], a “grey” zone appears in front of asymptomatic patients or case of patients presenting with discomfort or unexplained pain [2, 3, 5, 7, 8]. Generally considered a “minor procedure,” hardware removal is not complications-free, especially when upper limbs are involved [4, 6, 7, 9]. Indeed, several postoperative as well as intraoperative complications, especially technically related to implants, have been reported [4, 7, 10]. Since they seem to be associated also with the surgeon’s lack of experience [4, 7, 10], the literature suggests not leaving this surgery to residents without supervision [7, 10]. 

Without universal policies, literature tends to report orthopaedics and traumatology surgeons’ general tendencies and practices around countries and societies through the results of surveys and questionnaires [3–5, 10]. In this regard, a questionnaire performed in the 2006 AO Courses in Davos with 655 respondents from all around the world remarked a tendency of denial of routine implant removal in younger asymptomatic patients less than 40 years of age, but with a 68.9% agreement that it could represent a therapeutic choice in case of functional deficit or otherwise unexplained pain [5]. Indeed, in the case of symptomatic patients, the literature suggests improvements in function and decreased pain, with high rates of patient satisfaction, even when they encountered complications [11, 12]. Moreover, pain related to implants seems to impact intensely global dysfunction [12].

Lastly, with new materials and plates, survey results show that the removal of titanium implants is more challenging than stainless steel, mainly due to its mechanical properties [13].

This study aimed to describe Italian surgeons’ different attitudes, practices and beliefs regarding implant removal through a survey and compare them with the current international scenario.

## Materials and methods

### Study design and survey distribution

A Survey was conducted via an online Google format and distributed to all the Italian Orthopedics and Traumatologists registered to the Italian Society of Orthopedic Surgery and Traumatology (SIOT). The SIOT includes all Orthopedics and Traumatologists, residents and specialists of all ages, who facultatively register for it every year. The survey questionnaire was available from July 2024 to October 2024; each respondent could compile it anonymously once.

The aim was to gather information regarding attitudes, usual practices, beliefs and fears concerning implant removal. Participation was anonymous and voluntary with consent meant through submission.

### Questionnaire formulation and sources

A questionnaire of 25 items was realized based on the surveys concerning implant removal available in other countries and societies [3–5, 10, 11]. The questionnaires, retrieved from the appendix of each paper or directly from the documents, were scrutinized and analyzed. After modification, a new panel of questions was conceived. The survey format comprised a brief introduction with the survey’s intent and a paragraph with inclusion and exclusion criteria.

Inclusion criteria were healed fracture or osteotomy, full removal “in toto” of all osteosynthesis devices and removal of plates, nails, screws and cerclage wires or tension bands.

In agreement with the literature, paediatric patients < 16 years of age were excluded [2, 10, 14], as well as the removal of the single syndesmotic screw fixation and all the conditions which were collectively thought for implant removal as in case of nonunion/malunion, implant failure (as nail cut-out), allergy or metallosis, implant exposure or breakage and infection [2, 5]. The survey included general questions with a subdivision in the upper and lower limbs and more specific questions. According to the items, some had only one answer; others were multiple-choice questions with up to three answers possible. To indicate the percentage of removal, four groups were made with the following answer options: “0–20%”, “20%-50%”, “50%-80%”, “80%-100%”. The questionnaire was completed by the respondents if all the questions were validated. The questionnaire in the English version is available as Supplementary material 1.

### Data analysis

All the answers were grouped into an Excel spreadsheet, and a descriptive analysis was performed of all the responses with basic statistical measures. The analysis was made with exploratory purpose and without a null hypothesis base.

### Ethical consideration

The online spreadsheet ensured anonymity with no respondent identification. The survey was conducted in accordance with the declaration of Helsinki, and it does not describe experimental studies, and it did not need ethical approval. Respondents gave consent for study participation when anonymously answering the questionnaire. The questionnaire was facultatively compiled, and no financial awards were offered for its fulfillment.

## Results

A total of 500 answers were received and included in the descriptive analysis. Regarding asymptomatic patients, surgeons were predominantly reluctant to remove fixation devices (range 0–20%) in both the lower limbs (50.2%) and especially in the upper limbs (72.4%). In the younger asymptomatic group aged 16–40, despite a decrease in percentage in the range of 0–20% with 47.4% in the upper limbs and 30.6% in the lower limbs and a greater increase in removal, the trend of favoring non-removal was still confirmed. In symptomatic patients, the numbers changed significantly. A preference for removing fixation devices in 80–100% of cases was observed in the upper limbs (49.8%) and even more in the lower limbs (55.0%).

**Fig. 1:**
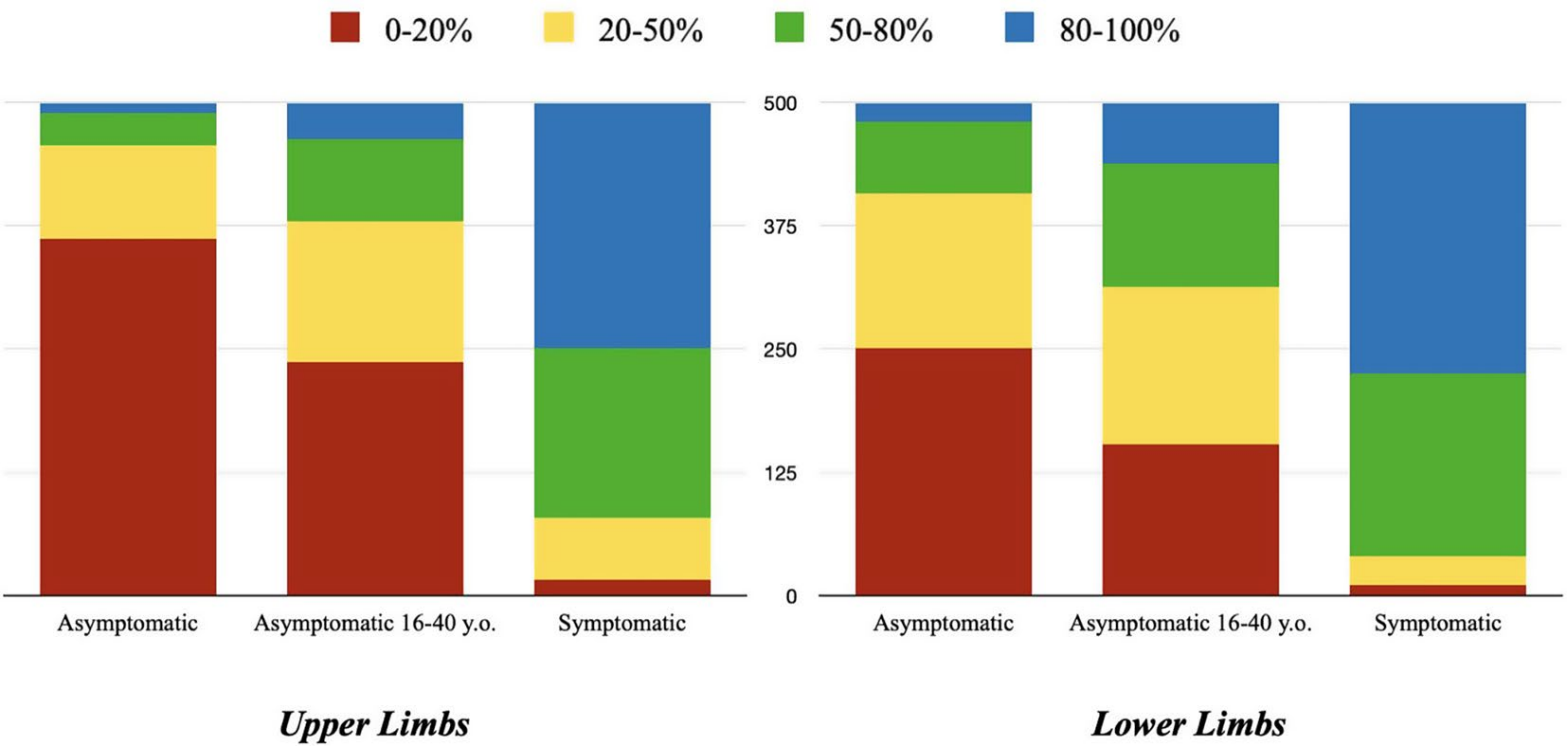
Percentages of asymptomatic patients, younger 16–40 y.o. asymptomatic patients and symptomatic patients undergoing implant removal in the upper and lower limbs

Regarding the most frequently removed implant in the upper limbs, the olecranon cerclage wire/tension band predominates (42.6%). In the lower limb, the distal fibular plate was the most removed device (53.6%). Upper and lower limbs results are reported in Tables 1 and 2 as well as in Fig. 1.

**Table 1:** Implants removal in the upper limbs

Upper Limbs	
In which percentage do you remove the fixation devices in asymptomatic patients, regardless of age?	n (%)
0–20%	362 (72.4%)
20–50%	94 (18.8%)
50–80%	33 (6.6%)
80–100%	11 (2.2%)
In which percentage do you remove the fixation devices in asymptomatic patients aged 16–40?	n (%)
0–20%	237 (47.4%)
20–50%	142 (28.4%)
50–80%	84 (16.8%)
80–100%	37 (7.4%)
In which percentage do you remove the fixation devices in symptomatic patients?	n (%)
0–20%	16 (3.2%)
20–50%	63 (12.6%)
50–80%	172 (34.4%)
80–100%	249 (49.8%)
Which segment had the highest number of device removals in the upper limbs?	n (%)
Proximal ulna	143 (28.6%)
Distal radius	115 (23.0%)
Proximal humerus	62 (12.4%)
Clavicle	55 (11.0%)
Radial shaft	38 (7.6%)
Distal humerus	36 (7.2%)
Carpus	16 (3.2%)
Ulnar shaft	12 (2.4%)
Proximal radius	11 (2.2%)
Humeral shaft	8 (1.6%)
Distal ulna	4 (0.8%)
In your experience, which device did you remove the most among the following?	n (%)
Olecranon cerclage wire/tension band	213 (42.6%)
Distal radius plate	91 (18.2%)
Clavicle plate	73 (14.6%)
Radial-ulnar shaft plates	49 (9.8%)
Proximal humerus plate	36 (7.2%)
Humeral nail	25 (5.0%)
Distal humerus plate	11 (2.2%)
Humeral shaft plate	2 (0.4%)

**Table 2:** Implants removal in lower limbs

Lower Limbs	
In which percentage do you remove the fixation devices in asymptomatic patients, regardless of age?	n (%)
0–20%	251 (50.2%)
20–50%	157 (31.4%)
50–80%	72 (14.4%)
80–100%	20 (4.0%)
In which percentage do you remove the fixation devices in asymptomatic patients aged 16–40?	n (%)
0–20%	153 (30.6%)
20–50%	160 (32.0%)
50–80%	125 (25.0%)
80–100%	62 (12.4%)
In which percentage do you remove the fixation devices in symptomatic patients?	n (%)
0–20%	11 (2.2%)
20–50%	29 (5.8%)
50–80%	185 (37.0%)
80–100%	275 (55.0%)
Which segment had the highest number of device removals in the lower limbs?	n (%)
Distal fibula	226 (45.2%)
Distal tibia	90 (18.0%)
Patella	42 (9.8%)
Proximal tibia	40 (8.0%)
Proximal femur	30 (6.0%)
Tibial shaft	27 (5.4%)
Femoral shaft	12 (2.4%)
Distal femur	11 (2.2%)
Proximal fibula	10 (2.0%)
Calcaneus	5 (1.0%)
Fibular shaft	4 (0.8%)
Talus	3 (0.6%)
In your experience, which device did you remove the most among the following?	n (%)
Fibular plate	268 (53.6%)
Patella cerclage wire/tension band	77 (15.4%)
Tibial plate	63 (12.6%)
Tibial nail	35 (7.0%)
Cervical-diaphyseal nail, DHS, PCCP for proximal femur fractures	32 (6.4%)
Nail for femoral shaft fractures	16 (3.2%)
Nail/plate for distal femur	9 (1.8%)

**Fig. 2:**
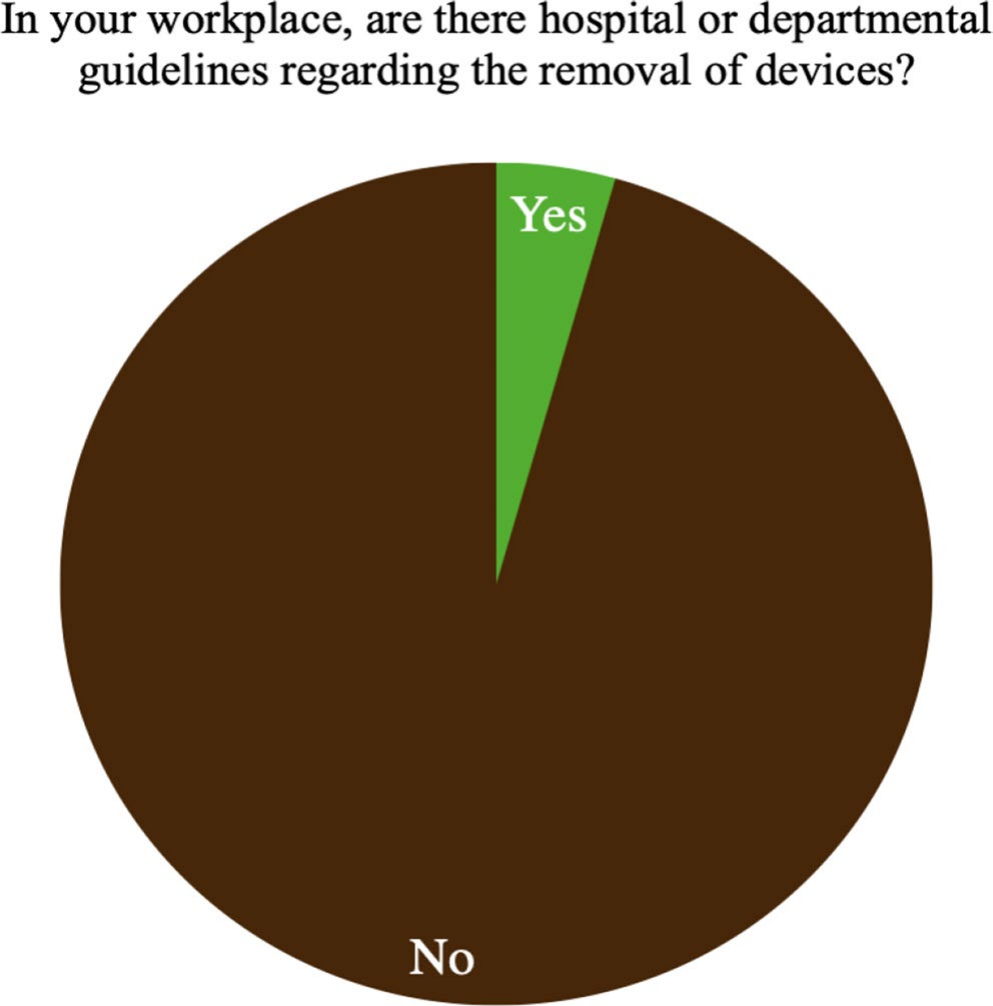
Existence of hospital guidelines in the Italian scenarios

**Fig. 3:**
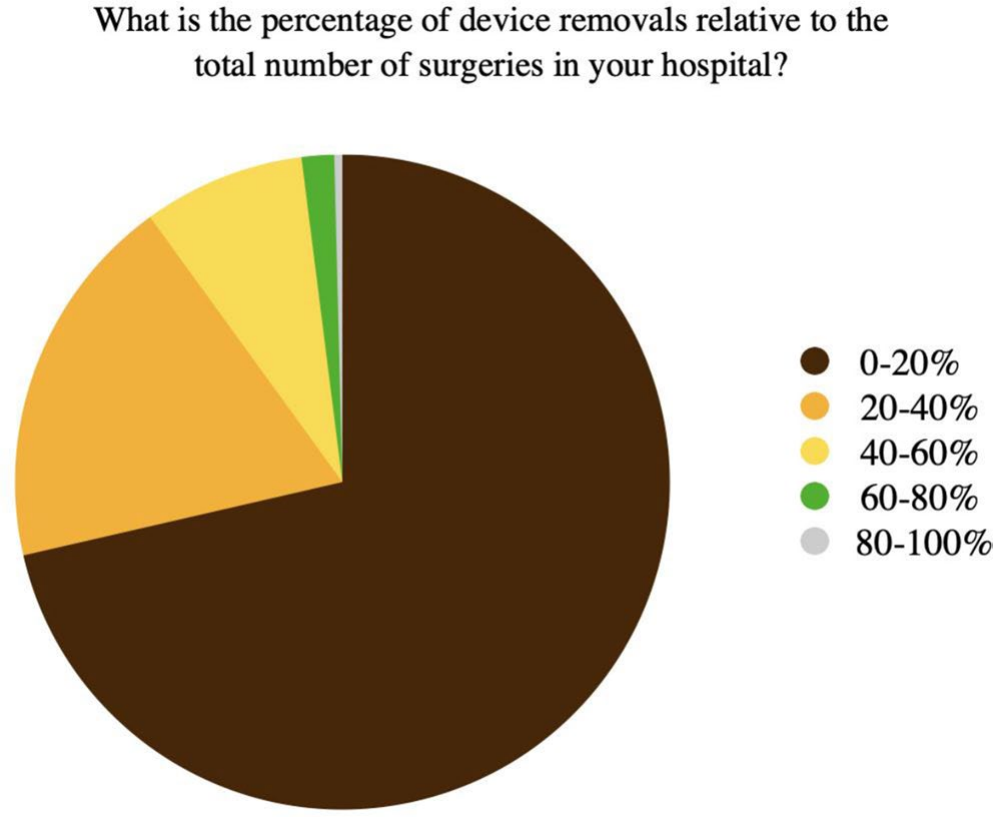
Percentages of implant removal relative to the total number of orthopedic surgical procedures per each respondent’s hospital

Of the 500 physicians interviewed, only 22 (4.4%) reported the presence of guidelines regarding implant removal in their facilities (Table 3) (Fig. 2), and 71.4% stated that the ratio of removal procedures was less than 20% relative to the total number of surgeries in their hospitals (Table 3) (Fig. 3).

**Table 3:** General responses concerning implant removal about the time from the initial surgery, the suitability for residents, existence of hospital guidelines and number of removals per hospital

General topics	
After how many months from the initial surgery do you remove the implant?	n (%)
< 6 months	0 (0%)
6–12 months	112 (22.4%)
12–18 months	311 (62.2%)
18–24 months	67 (13.4%)
> 24 months	10 (2.0%)
Do you think implant removal to be a procedure suitable for a resident?	n (%)
Yes	113 (22.6%)
No	387 (77.4%)
Question reserved for residents: How self-confident do you feel in performing it, considering supervision? (1 = too little − 10 = too much) (95 responses)	n (%)
1	2 (2.1%)
2	0 (0%)
3	3 (3.2%)
4	2 (2.1)
5	5 (5.3%)
6	11 (11.6%)
7	19 (20.0%)
8	24 (25.3%)
9	16 (16.8%)
10	13 (13.7%)
In your workplace, are there hospital or departmental guidelines regarding the removal of devices?	n (%)
Yes	22 (4.4%)
No	478 (95.6%)
What is the percentage of device removals relative to the total number of surgeries in your hospital?	n (%)
0–20%	357 (71.4%)
20–40%	93 (18.6%)
40–60%	40 (8.0%)
60–80%	8 (1.6%)
80–100%	2 (0.4%)

Among the 500 physicians interviewed, 81.0% were specialists, while 19.0% were residents, with most of them reporting a high level of self-confidence in implant removal (Table 3).

Concerning timing, Italian orthopaedic surgeons prefer to wait between 12 and 18 months before proceeding with implant removal (62.2%), and, in any case, at least six months from the initial surgery usually occur (Table 3).

A clear majority of respondents reported that the primary indication for removing implanted devices was specific patient discomfort (92.8%), followed by attempts to prevent subsequent complications (49.0%) and potential surgical challenges (38.2%). Many surgeons expect that removing implants may resolve issues such as pressure on the skin and soft tissues (78.4%), pain (58.8%), and ROM limitations (45.8%). At the same time, when performing this type of surgery, the most feared complication was the potential for hardware breakage and stripping of screws during the procedure (48.2%). These percentages were mirrored in the surgical field as the most frequently encountered complication was stripping of the head screws (67.2%), with only three participants (0.6%) reporting never having had problems.

Regarding postoperative outcomes, the most frequently reported issues included unpleasant scars (35.4%), persistence of symptoms (31.4%), and bleeding (26.6%).

Indications for removal, intraoperative and postoperative, most feared complications are reported in Table 4. When fixation device removal is performed for elective surgery such as prosthetic replacement, 50.8% of physicians prefer to adopt a one-stage protocol. On the other hand, those who opt for a two-stage approach are primarily concerned about the increased risk of infection (64.2%) and most of them wait three to six months between the two surgical procedures (59.8%) (Table 5).

**Table 4:** Main indications, reasons and expectations, intraoperative and postoperative complications concerning implant removals

Indications, expectations, intraoperative and postoperative complications	
What are your main indications/reasons for implants removal? (Maximum 3 responses)	n (%)
In case of specific discomfort	464 (92.8%)
To prevent future complications (example peri-implant breakage)	245 (49.0%)
To avoid future surgical issues	191 (38.2%)
Patient request	172 (34.4%)
To prevent late infection	25 (5.0%)
Bad experiences with implants in situ	15 (3.0%)
Personal preference	13 (2.6%)
No specific reason	12 (2.4%)
I was taught to remove them	2 (0.4%)
Which patient complaints do you expect to improve after removal? (Maximum 3 responses)	n (%)
Skin or soft tissues pressure issues	392 (78.4%)
Pain	294 (58.8%)
Limited range of motion (ROM) and proprioception	229 (45.8%)
Swelling and inflammation	143 (28.6%)
Paresthesia	36 (7.2%)
Intraoperatively, what complications do you encounter the most during implants removal? (Maximum 3 responses)	n (%)
Stripping of screw heads	336 (67.2%)
Implants welding/cold fusion	218 (43.6%)
Inability to fully remove the device	192 (38.4%)
Implant breakage	186 (37.2%)
Bone overgrowth	147 (29.4%)
Larger incision necessary	127 (25.4%)
Implant difficult to find	113 (22.6%)
Inadequate instruments for removal	111 (22.2%)
Surgery longer than expected	107 (21.4%)
Increased fluoroscopy usage	49 (9.8%)
Iatrogenic fractures	27 (5.4%)
Bleeding	22 (4.4%)
Nerve injuries	8 (1.6%)
I never had problems	3 (0.6%)
Postoperatively, which complications/outcomes do you encounter the most? (Maximum 3 responses)	n (%)
Aesthetic issues/ Unpleasant scar	177 (35.4%)
Persistence of symptoms	157 (31.4%)
Bleeding	133 (26.6%)
No complications	112 (22.4%)
Surgical site infection	69 (13.8%)
Refracture	58 (11.6%)
Nerve injuries	19 (3.8%)
Wound dehiscence	3 (0.6%)
Hematoma	1 (0.2%)
Delayed wound healing	1 (0.2%)
Independently of objective data and studies, what subjectively concerns you the most about implant removal?	n (%)
“What if I break the implant? What if I strip the screws?”	241 (48.2%)
“Will I have the right instruments?”	126 (25.2%)
“What if I cause an intraoperative fracture?”	45 (9.0%)
“I wasn’t the one who implanted it…”	40 (8.0%)
“What if it gets infected?”	26 (5.2%)
“Will the fracture be fully healed?”	22 (4.4%)

**Table 5:** One- versus two-stage protocol in case of replacement and main reasons

One- versus two-stage protocol	
In cases where elective surgery/replacement will be necessary in the presence of implants, do you opt for a one-stage protocol (removal and surgery in the same surgery) or a two-stage protocol (initial removal followed by elective surgery later)?	n (%)
One-stage	254 (50.8%)
Two-stage	246 (49.2%)
In the case of a two-stage protocol, what is the main reason for your choice? (246 responses)	n (%)
Increased risk of infection	158 (64.2%)
Complications related to device removal (e.g., loss of bone stock, unknown implant)	55 (22.4%)
Increased surgical time	17 (6.9%)
Need for two surgical accesses	16 (6.5%)
In the case of a two-stage protocol, how many months after implant removal do you perform the replacement? (246 responses)	n (%)
< 3 months	30 (12.2%)
3–6 months	147 (59.8%)
6–12 months	63 (25.6%)
> 12 months	6 (2.4%)

Finally, when analyzing differences in hardware materials, titanium implants are not considered safer to be left in situ (62.2%) but are regarded as more difficult to remove compared to stainless steel ones (62.0%) (Table 6).

**Table 6:** Materials’ influence on implant removal (Titanium versus stainless steel)

Materials (Titanium versus Stainless steel)	
Do you think titanium devices are “safer” to leave in situ compared to stainless steel devices?	n (%)
Yes	189 (37.8%)
No	311 (62.2%)
Are there any differences in the removal of titanium and stainless-steel devices?	n (%)
Titanium devices are more difficult to remove compared to stainless steel	310 (62.0%)
I have not found any difference between titanium and stainless steel	104 (20.8%)
Titanium devices are easier to remove compared to stainless steel	64 (12.8%)
I have no experience with these materials	22 (4.4%)

## Discussion

The most highlighted finding of this survey, in agreement with the current evidence, is a tendency not to routinely remove implants in asymptomatic patients, both in the lower as well as in the upper limbs. This inclination emerges also in younger patients. On the contrary, in the case of symptomatic patients, implant removal is considered a therapeutic choice. However, guidelines are lacking in almost all the Italian scenarios.

Often considered a minor procedure, implant removal could evolve into complex surgery. For this reason, it is not considered suitable for a resident without supervision, with the most feared intraoperative complications being stripping of the heads’ screws and implant breakage. In addition, concerning the introduction of new materials, titanium implants are thought to be more challenging to remove respect to stainless steel ones.

The percentage of surgeries for implant removal differs from state to state, from 5 to 30% of all elective procedures [1–4]. This survey results align with the international scenario since most respondents agreed that this surgery accounts for 0–20% of all surgeries performed in their centres.

This international practice and literature variety could be derived from a lack of universal guidelines [2–4].

While there are clear indications in case of infection, nonunion/malunion, implant breakage or failure [2, 5, 6], controversy exists in front of asymptomatic patients or cases of discomfort or otherwise unexplained pain [2, 3, 5, 7, 8]. The lack of evidence-based or hospital guidelines throughout the Italian territory reflects the international scenario [4]. Jamil et al., in the United Kingdom national survey, reported 90% not having guidelines, which approximates the 96% of this survey [4].

This study underlines that in the asymptomatic patients without age restriction, there is a general tendency not to remove the implant, both in the lower but with a higher percentage in the upper extremities. The same is shown in the literature by the study of Jamil et al., where 92% of respondents did not remove hardware in asymptomatic skeletally mature patients [4]. The main reason is that implant removal is not without intraoperative and postoperative complications [2–4, 10]. Postoperative complications vary from 0 to 30%, with the most mentioned being neurological lesions, refracture, infection and persistence of symptoms [2–4, 10]. The Dutch survey results reported 37% of infection, 24% of unpleasant scarring and 19% of postoperative hemorrhage [3].

Mingo-Robinet and Pérez Aguilar, in the Spanish survey, described 11% of neurological lesions, 17.7% of refractures and surgical wound infection in 11.6% of cases [10]. Moreover, they reported persistence of symptoms when already present in 39.8% of cases, which corresponds approximately to this survey percentage [10].

In addition, pain or symptoms can worsen or appear after hardware removal [5, 15]. Indeed, Gosling et al. reported 20% of complaints at follow-up when considering 51 asymptomatic patients who underwent femoral nail removal [15].

Most postoperative complications reported in this survey were entirely in agreement with the literature being scarring wound concerns, persistence of symptoms and bleeding.

Nonetheless, Ko et al. reported a significant improvement in the quality of life and satisfaction after implant removal even in asymptomatic patients [16].

When considering the younger group of asymptomatic patients with a range of age 16–40, still a tendency of not implant removal has emerged, in agreement with the current evidence [3–5].

Hanson et al. reported that the majority of surgeons did not agree that implants should be removed in younger asymptomatic patients [5]. However, in this survey they were removed with higher percentages with respect to the general group. The main reasons reported are two [3, 10, 17]: the likely increased difficulty in subsequent reconstruction/arthroplasty surgery and the risk of peri-implant fracture in case of return to sports activity [3, 10]. Concerning the first, bone loss and diminished stock or augmented infection risk were the main causes, leading sometimes to the necessity of a two-stage procedure [10].

Concerning the risk of peri-implant fracture with the return to sports, the literature agrees there is no evidence of hardware removal before the resumption of sports [4, 10]. In this regard, Evans and Evans evaluated 15 professional rugby athletes who resumed contact sports with hardware in situ, with only two suffering complications and 13 without complications up to six years after surgery [18]. Thus, the Authors suggested that avoiding delays associated with implant removal in professional athletes leads to minimal absence from competitive participation and prevents economic defeats [18].

In addition, one of the latest papers published by Acklin et al. suggests that contact activities can be resumed with the implant in places since when the implant is withdrawn, incomplete filling of the plate hollows last for 18 months, with a suggested abstention from contact sport of at least four months [7]. However, when considering peri-implant fracture, the literature mentions a higher risk in plate peripheral screw holes with respect to intramedullary nails [7].

What has emerged from the literature is that the risk of malignancy or carcinogenesis with retained metal implants is extremely rare [4, 5]. For example, Jamil et al. reported that 87% of practicing surgeons believe leaving the implants in situ for ten years or more is possible since the co-occurrence of a malignancy development is more feasible with respect to a causal effect [4]. The same emerged from the AO survey by Hanson et al. [5]. Moreover, while allergy to stainless steel and its components such as nickel or chromium has been reported, allergy to titanium and its metal alloys is less frequent, and it is still debated whether true contact allergy to pure titanium exists [7].

An exclusion criterion throughout the survey that has been applied was hardware removal in pediatric patients less than 16 years of age. This since the literature reports a tendency for routine implant removal in children [2, 3, 10].

For example, in the Spanish survey, 31% of respondents always remove implants in children [10], and up to 72% of surgeons in the Dutch study remove elastic nails by default [3].

However, there is controversy concerning the paediatric population, especially between paediatric and nonpaediatric surgeons [10, 14]. While nonpaediatric specialists tend to be more prone to implant removal due to the possible challenges in future extraction or reconstruction, paediatric surgeons are more reluctant to remove asymptomatic hardware [10, 14].

A different chapter concerns symptomatic patients presenting with pain or functional deficits. In this scenario, most of the respondents of this survey will remove implants, with, however, more numerous percentages in the lower limbs with respect to the upper limbs. This tendency agrees with Hanson et al. [5] and Acklin et al. [7]. The Dutch survey revealed it as a good option when symptoms are present [3].

Apart from surgeon surveys, patient surveys have been realized to evaluate the outcomes [11]. Reith et al., after hardware removal, reported an improvement in function of 72% and decreased pain in 96% of patients who responded to the questionnaire [11]. Patient satisfaction after hardware removal was high, with 96% of all patients and 66% of those with postoperative or perioperative complications that would opt for surgery again [11]. Kempton et al. underlined how implant-related pain contributes to patient global dysfunction since patients starting with worse global indices were more likely to improve after the removal [12]. Moreover, the surgeon’s expectation of pain improvement was accurate 82% of the time overall, 84% of the time when predicting advancement, and 50% when predicting no improvement [12]. Since this last percentage, the Authors suggested that it may be valid to undertake surgery even if the surgeon doubts that it will improve the symptoms, especially when implant removal is a last resort to improve pain [12].

A brief parenthesis is deserved for the upper limbs. As mentioned, implant removal in the upper limbs is in all the categories analyzed less frequently than in lower limbs, even in the case of symptoms. This trend aligns with the literature explaining it, especially concerning the forearm, with a higher refracture rate and potential damage to neurovascular structures such as the posterior interosseous nerve after plate removal [4, 9]. When considering olecranon, distal radius and clavicle, Hambrecht et al. reported hardware removal as a safe procedure but with a lower patient satisfaction rate when indications are established on unspecific symptoms of discomfort [6].

Concerning the time from the first surgery to implant removal, 62.2% of respondents performed it between 12 and 18 months. None remove the implants before six months. This percentage is similar to the Spanish survey, where 64.4% waited more than 12 months before removal [10].

When considering the upper limbs, the respondents to the survey agreed that the most removed implants are the olecranon tension band, the plate for the distal radius and the clavicular plate. This result aligns with the international trends as reported by Mingot-Robinet and Pérez Aguilar [10] and Vos et al. [3] describing the clavicle plate and the olecranon tension band as the most extracted devices.

However, in contrast to current literature [3, 5, 10], the most common osteosynthesis device removed from the lower limbs in this survey was the fibular plate instead of the patella tension band. Indeed, the patella tension band seems to be the most removed implant in the international scenario [3, 5, 10].

The rationale for this difference could derive from two main elements. The first is epidemiological since patellar fractures are increasing in elderly patients as part of the fragility fractures [19, 20]. Still, in this survey, most implants are removed in younger patients, so the survey focuses more on fractures occurring in younger or more active patients.

Secondly, considering the survey questions regarding the indications for implant removal, one of the main reasons was discomfort. When considering the expected improvements, most answers concerned skin and soft tissue pressure issues. So, by intermingling all the data, the fibular plate could be predicted to be the most removed in the SIOT survey.

In the survey distributed to patients to evaluate their point of view, most of the implants removed were at the ankle joint (21%) [11]. As reported by Busam et al., results are contrasting regarding pain improvement after implant removal for ankle fractures that underwent ORIF [2]. In addition, concerning the implants, increased use and popularity of mini-fragment plates for lateral malleolar fixation have emerged [21]. However, Swenson et al. showed that elective implant removal rates were similar between mini-fragments and small-fragment fixation of distal fibular fractures [21].

Moreover, recently, with early weight-bearing after ORIF for ankle fractures, Rockov et al. reported a similar percentage of implant removal for pain or prominence both in the early and late weight-bearing group [22].

Even if frequently considered a minor procedure [4], 77% of respondents agree that this is not a surgery for residents. Indeed, it could evolve from simple to complex, as reported in the current literature [4, 7, 23].

Interestingly, Page et al. reported that all the removals, but two patients, were performed by the same senior surgeons who performed the first surgery [24]. This would have simplified the removal since the surgeons knew the prior surgical field and the implants [24].

However, when specifically questioned the residents about their self-confidence on implant removal on a scale from 1 to 10, in 76% of cases they felt confident ≥ 7.

This result was justified by the Authors of this survey with the following: firstly, according to the Italian guidelines in every center, the resident has the possibility and the obligation to work supervised and assisted by a senior doctor; secondly, because being the survey addressed to participants registered in the SIOT, the ones enrolled are probably already towards the end of the residency program respect to the beginning. Indeed, implant removal is not a “resident surgery”, without supervision, as reported by the literature, because it could evolve into a complex procedure [4, 7]. In the Spanish Survey, 0% of extraction procedures were performed by a resident without supervision [10]. The same is reported by the Dutch study, where even if implant removal is considered by more than half of the respondents a procedure suitable for junior residents, 90% agree that this procedure is more appropriate for senior residents [3].

When asked which is the most feared complication, respondents agreed with the fear of breaking the implant, stripping the screw heads and the lack of adequate instrumentation for removal. The infection risk or if the fracture would be unhealed revealed marginal roles throughout this survey.

The most feared complications were mirrored by the true encountered intraoperative complications: stripping of the screw heads, cold fusion and inability to remove the implants in toto.

These results are in line with the current literature where most intraoperative complications encountered are the surgery longer than expected due to bone overgrowth and implants challenging to find, stripping of the screws, cold fusion, and enlargement of the original surgical incision [3, 10]. Only 3 out of 500 participants reported no intraoperative complications in the SIOT survey.

Acklin et al. describe how stripping of the screw in the locking plates is a significant problem during removal [7]. The difficulty depends on the screw size, with the smaller ones at greater risk, on the location since metaphyseal regions are more complex than diaphyseal and on the material, with titanium being at more significant risk for stripping than stainless steel [7, 9, 25, 26].

In order to avoid complications or, in the end, the risk of not being able to remove the implant, Mingo-Robinet and Pérez Aguilar mentioned the availability of a specific material extraction box available at the hospital, however available for only 26% of the Spanish survey respondents [10].

As mentioned, the most answered indications for implant removal were patients’ discomfort and the avoidance of possible future complications.

When considering the expectations, the main ones were the pressure improvement on the skin and soft tissues, pain reduction and ROM amelioration. All these perfectly deal with the current literature [3, 10].

Regarding the material, 62% of the considered titanium implants are more demanding to remove with respect to stainless steel. This percentage corresponds to the Spanish [10] and the Dutch [3] surveys, where approximately 62% of the respondents agree with the easier removal of stainless steel implants. The reason is the material properties since titanium is softer than stainless steel, giving the surgeons different tactile feedback [7, 13, 27]. Despite troubles with titanium implants, Dehghan et al. documented a low rate of screw breakage, stripping and cold fusion during the removal, with nonetheless, 7.6% of the surgeries requiring extra instruments and operative time [13].

Since one of the reasons for implant removal in younger patients is future reconstruction/arthroplasty, one question regards removal and arthroplasty in one or two stages. The answers were approximately 50% and 50%, with the main reason to adopt a two-stage protocol being avoiding infection risk. According to the interval, most respondents perform the replacement between three and six months from implant removal. This is a debated topic in literature, with some Authors advocating one or two-stage approach [10, 28–33].

For example, Baker et al. recently described hardware removal accomplished together or within three months of a TKA as being associated with increased odds of periprosthetic joint infection at one year [28]. Thus, they suggested removing the hardware before TKA [28].

Table 7 is a summary of the main differences and similarities with international trends.

**Table 7:** Summary of the main differences and similarities between the SIOT survey and international trends. % = percentage; rom = range of motion; ss = stainless steel. * Considered as exclusion criteria for the SIOT survey. Inclusion and exclusion criteria of the SIOT survey are applied

	SIOT survey	International trends
Most frequent percentage of implant removal in asymptomatic patients (regardless of the age - upper and lower limbs)	0–20% (Upper limbs) 0–20% (lower limbs)	(In the following studies not division upper and lower limbs) • 92% of respondents do not routinely remove implants [4] • “…overall tendency against routine metal removal…” [5]
Most frequent percentage of implant removal in younger asymptomatic patients (16–40 years old – upper and lower limbs)	0–20% (Upper limbs) 20–50% (Lower limbs)	(In the following studies not division upper and lower limbs) • 34% of respondents agreed to remove [3] • 37.1% of respondents agreed to remove [5] • 28% of respondents agreed to remove [10]
Most frequent percentage of implant removal in symptomatic patients (upper and lower limbs)	80–100% (Upper limbs) 80–100% (Lower limbs)	(In the following studies not division upper and lower limbs) • 89% of respondents agreed to remove [3] • 68.9% of respondents agreed to remove [5] Concerning the upper limbs: • Lower rate of patient satisfaction when indications based on unspecific symptoms of discomfort [6]
Most frequent implant removed (upper limbs)	Olecranon cerclage wire/tension band	• Olecranon tension band [3] • Routinely removal for internal fixation of the clavicle [4] • Cerclage wire olecranon [5] • Olecranon tension band [10]
Most frequent implant removed (lower limbs)	Fibular plate	• Patella tension band [3] • Routinely removal for midshaft tibial fractures [4] • Cerclage wire patella [5] • Kneecap tension band [10]
Most frequent intraoperative complications	Stripping of screw heads, implants welding/cold fusion, inability to fully remove the device, … 0.6% never had problems	• Bony overgrowth, surgery longer than planned, enlargement of the original incision, implant difficult to find, stripping of screw heads and cold welding, … 4% no problems observed. [3] • Bone overgrowth, stripping of the screw heads, surgery longer than planned, impossibility of removing part of complete implants, … 0% never had problems [10]
Presence of hospital guidelines (yes/no and %)	No (95.6%)	• Lack of policy guidelines … [3] • No (90%) [4] • “… Disparity of treatment guidelines…” [10]
Most frequent percentage of implant removal relative to the total number of surgeries in your hospital	0–20%	• Removal procedures account for 29% of all elective operations and for 15% of all operations at the department [1]
Most frequent postoperative complications	Aesthetic issues/ Unpleasant scar, persistence of symptoms, bleeding, … 22.4% no complications	• Wound infection, unpleasant scarring (24%) and postoperative hemorrhage, … 13% no complications [3] • Persistence of symptoms, unsatisfactory scar, refracture, …19.5% no complication [10]
Most common time (months) from initial surgery to implant removal (%)	12–18 months (62.2%)	• Upper extremities 6–12 months (46%), lower extremities 12–18 months (49%) [3] • 6–12 months (31.7%); > 12 months (64.4%) [10]
Procedure for resident (yes/no and %)	No (77.4%)	• 65% suitable for junior resident, 90% agreed more suitable for senior residents [3] • 0% procedures by resident without supervision [10]
Most common indications for implant removal	In case of specific discomfort, to prevent future complications, to avoid future surgical issues, …	• Infection*, in case of specific patient complaints, on patient’s request, … [3] • Localized pain, loosening*, infection*, metalwork damage*, skin irritation, … (not ordered by frequency) [4] • Palpable/irritating material, pain, limited ROM, … [5] • Specific discomfort, infection*, implant rupture*, on patient’s request, to avoid future surgical problems, … [10]
Expectations after implant removals	Skin or soft tissues pressure issues, pain, limited ROM and proprioception, …	• Skin or soft tissue pressure issues, pain, limited ROM, swelling, … [3] • Skin or soft tissue pressure issues, pain, limited joint balance, … [10]
Titanium versus Stainless Steel (SS) implants (%) – Difficulty in implant removal	Titanium more difficult (62%)	• Titanium more difficult (62%) [3] • SS easier (62.8%) [10]

This study encompasses some limitations.

Firstly, since it is a survey, it describes the practices and attitudes of surgeons, and it does not provide guidelines or evidence. So, it should not be considered a guideline, but the Authors’ intention was purely descriptive. Then, being a survey distributed within the SIOT, only surgeons registered to the SIOT could participate. However, this last point has the advantage of including residents and specialists working in different hospital settings, public, university and private hospitals, thus reducing the bias of describing the practice applied in only one type of hospital.

Lastly, even if the Authors extrapolated questions from the other surveys to make this survey as comprehensive and straightforward as possible, some questions could be considered vague and prone to bias.

Future high-quality or extensive multicenter studies will be necessary to identify more homogenous guidelines and to standardize the current practice.

## Conclusion

With the lack of universal guidelines throughout the Italian scenario, this study describes a general tendency not to routinely remove hardware, even in younger patients, unless in case of symptoms where implant removal could be considered a therapeutic option.

Indeed, implant removal should not be underestimated and regarded as a simple surgery since it is not freed from postoperative as well as intraoperative complications such as stripping of the screws.

## Electronic supplementary material

Below is the link to the electronic supplementary material.

Supplementary Material 1

## Data Availability

Data is provided within the manuscript or supplementary information files.

## Abbreviations

SIOT: Italian Society of Orthopedic Surgery and Traumatology (SIOT)
AO: Arbeitsgemeinschaft für Osteosynthesefragen
ROM: Range of motion
ORIF: Open reduction and internal fixation
TKA: Total knee arthroplasty
y.o: Years old
n: Numbers
