# Correction to: Implant removal: benefits and drawbacks - Results of a survey with five hundred participants from the Italian Society of Orthopedic Surgery and Traumatology (SIOT) and comparison with other international trends

**DOI:** 10.1007/s00264-025-06583-4

**Published:** 2025-06-18

**Authors:** Virginia Masoni, Corrado Ciatti, Luca Andriollo, Giovanni Vicenti, Fabrizio Rivera

**Affiliations:** 1 University of Turin, Turin, Italy; 2 Guglielmo da Saliceto Hospital, Piacenza, Italy; 3 University of Parma, Parma, Italy; 4 Fondazione Poliambulanza Istituto Ospedaliero, Brescia, Italy; 5 Catholic University of the Sacred Heart, Milan, Italy; 6 University of Bari Aldo Moro, Bari, Italy; 7 Ospedale SS Annunziata, ASL CN1, Savigliano, Italy

**Correction to: international orthopaedics**

10.1007/s00264-025-06564-7

1) In the “Introduction” section of the abstract, a paragraph from another article was mistakenly reported during the editing process. The article part included by mistake refers to the abstract introduction of the following: Belyea CM, Rummel G, Lanham N, Krul KP. Upper Extremity and Musculoskeletal Injuries Related to Videogame and Augmented Reality Game Usage: A Narrative Review Article. Curr Rev Musculoskelet Med. 2025 May;18(5):201-206. doi: 10.1007/s12178-025-09956-9. Epub 2025 Feb 13.PMID: 39946075; PMCID: PMC12014968.

The correct Abstract Introduction paragraph, should be:

Implant removal in orthopaedics and traumatology is still a controversial topic. Benefits and drawbacks lead to relative indications, mainly depending on patients’ demands and surgeons’ perspectives. This study aims to report the current attitudes and practices of Italian surgeons who participated in a survey. And not the previously reported Since the introduction of videogames and augmented reality technology, injuries associated with e sports have garnered increased attention from researchers and healthcare professionals. This review articles examines the spectrum of injuries associated with videogames and augmented reality and describes the nuances of the diagnoses associated with gaming injuries.

2) Fabrizio Rivera correct affiliation is Ospedale SS Annunziata, ASL CN1, Savigliano, Italy and not Ospedale SS. Annunziata, Chieti, Italy.

3) The two authors Virginia Masoni and Corrado Ciatti share the first Coauthorship.

The original article has been corrected.

