# Supplementary material for: Implant removal: benefits and drawbacks - Results of a survey with five hundred participants from the Italian Society of Orthopedic Surgery and Traumatology (SIOT) and comparison with other international trends
Source: Int Orthop. 2025 May 26;49(8):1775–87. doi: 10.1007/s00264-025-06564-7 (PMC12283862; doi:10.1007/s00264-025-06564-7)
Supplement: Supplementary file 1 — Supplementary Material 1 [file 264_2025_6564_MOESM1_ESM.docx]

**Supplementary Material.1**

**The version below is the English translation of the original questionnaire distributed in Italian language through Google Format. In the English language the words implants and devices are used as synonyms.**

Inclusion Criteria

1. Healed fracture/osteotomy
2. Complete (in toto) removal of the implant
3. The implants or devices considered are plates, nails, screws and cerclage wires/tension band

Exclusion criteria

1. Removal of the single syndesmotic screw
2. Age < 16 years old
3. Complications or proved failures of the implants (nonunion, implant breakage, infection, implant allergy/metallosis, implant exposure/soft tissue concerns, early osteosynthesis failure as nail cut-out)

1) In which percentage do you remove the fixation devices in asymptomatic patients, regardless of age? (Upper limbs)

· 0-20%

· 20-50%

· 50-80%

· 80% - 100%

2) In which percentage do you remove the fixation devices in asymptomatic patients, regardless of age? (Lower limbs)

· 0-20%

· 20-50%

· 50-80%

· 80% - 100%

3) In which percentage do you remove the fixation devices in asymptomatic patients aged 16-40? (Upper limbs)

· 0-20%

· 20-50%

· 50-80%

· 80% - 100%

4) In which percentage do you remove the fixation devices in asymptomatic patients aged 16-40? (Lower limbs)

· 0-20%

· 20-50%

· 50-80%

· 80% - 100%

5) In which percentage do you remove the fixation devices in symptomatic patients? (Upper limbs)

· 0-20%

· 20-50%

· 50-80%

· 80% - 100%

6) In which percentage do you remove the fixation devices in symptomatic patients? (Lower limbs)

· 0-20%

· 20-50%

· 50-80%

· 80% - 100%

7) After how many months from the initial surgery do you remove the implant?

· < 6 months

· 6-12 months

· 12-18 months

· 18-24 months

· >24 months

8) Do you think implant removal to be a procedure suitable for a resident?

· Yes

· No

9) Question reserved for residents: How self-confident do you feel in performing it, considering supervision? (1 = too little - 10 = too much)

· 1

· 2

· 3

· 4

· 5

· 6

· 7

· 8

· 9

· 10

10) Which segment had the highest number of device removals in the upper limbs?

- Clavicle
- Proximal humerus
- Humeral shaft
- Distal humerus
- Proximal radius
- Proximal ulna
- Radial shaft
- Ulnar shaft
- Distal radius
- Distal ulna

· Carpus

11) Which segment had the highest number of device removals in the lower limbs?

· Proximal femur

· Femoral shaft

· Distal femur

· Proximal tibia

· Proximal fibula

· Tibial shaft

· Fibular shaft

· Distal tibia

· Distal fibula

· Patella

· Talus

· Calcaneus

12) In your experience, which device did you remove the most among the following? (Upper limbs)

- Clavicle plate
- Distal humerus plate
- Proximal humerus plate
- Humeral shaft plate
- Humeral nail
- Olecranon cerclage wire/tension band
- Radial-Ulnar shaft plates
- Distal radius plate

13) In your experience, which device did you remove the most among the following? (Lower limbs)

- Cervical-diaphyseal nail, DHS, PCCP for proximal femur fractures
- Nail for femoral shaft fractures
- Nail/plate for distal femur
- Tibial nail
- Tibial plate
- Fibular plate
- Patellar cerclage wire/tension band

14) In your workplace, are there hospital or departmental guidelines regarding the removal of devices?

- Yes
- No

15) What are your main indications/reasons for implants removal? (Maximum 3 responses)

- No specific reason
- I was taught to remove them
- Patient request
- In case of specific discomfort
- To avoid future surgical issues
- To prevent future complications (example peri-implant breakage)
- To prevent late infection
- Bad experiences with implants in situ
- Personal preference

16) Which patient complaints do you expect to improve after removal? (Maximum 3 responses)

- Skin or soft tissues pressure issues
- Pain
- Paresthesia
- Swelling and inflammation
- Limited ROM and proprioception

17) Intraoperatively, what complications do you encounter the most during implants removal? (Maximum 3 responses)

- - I never had problems
  - Nerve injuries
  - Increased fluoroscopy usage
  - Bleeding
  - Iatrogenic fractures
  - Larger incision necessary
  - Implant difficult to find
  - Implants welding/cold fusion
  - Inadequate instruments for removal
  - Implant breakage
  - Inability to fully remove the device
  - Surgery longer than expected
  - Stripping of screw heads
  - Bone overgrowth

18) Postoperatively, which complications/outcomes do you encounter the most? (Maximum 3 responses)

- No complications
- Persistence of symptoms
- Aesthetic issues/ Unpleasant scar
- Refracture
- Bleeding
- Surgical site infection
- Nerve injuries
- Others: __________________________

19) In cases where elective surgery/replacement will be necessary in the presence of implants, do you opt for a one-stage protocol (removal and surgery in the same surgery) or a two-stage protocol (initial removal followed by elective surgery later)?

- One-stage
- Two-stage

20) In the case of a two-stage protocol, what is the main reason for your choice?

- Need for two surgical accesses
- Increased surgical time
- Complications related to device removal (e.g., loss of bone stock, unknown implant)
- Increased risk of infection

21) In the case of a two-stage protocol, how many months after implant removal do you perform the replacement?

- < 3 months
- 3-6 months
- 6-12 months
- 12 months

22) Independently of objective data and studies, what subjectively concerns you the most about implant removal?

- "Will I have the right instruments?"
- "What if I break the implant? What if I strip the screws?"
- "What if it gets infected?"
- "What if I cause an intraoperative fracture?"
- "Will the fracture be fully healed?"
- "I wasn’t the one who implanted it..."

23) Do you think titanium devices are "safer" to leave in situ compared to stainless steel devices?

- Yes
- No

24) Are there any differences in the removal of titanium and stainless-steel devices?

- I have no experience with these materials
- I have not found any difference between titanium and stainless steel
- Titanium devices are easier to remove compared to stainless steel
- Titanium devices are more difficult to remove compared to stainless steel

25) What is the percentage of device removals relative to the total number of surgeries in your hospital?

- 0-20%
- 20-40%
- 40-60%
- 60-80%
- 80-100%
